# The Association between Food Insecurity and Academic Performance among Higher Education Students: A Systematic Review

**DOI:** 10.1007/s13668-026-00744-6

**Published:** 2026-02-28

**Authors:** Prosper M. Galseku, Gregory S. Keenan, Leo Stevenson, Rosanna Cousins, Jamie Lingwood, Hannah Dixon, Lorna Bourke

**Affiliations:** 1 School of Psychology, Liverpool Hope University, Liverpool, L16 9JD UK; 2 School of Sport and Health Sciences, Liverpool Hope University, Liverpool, UK; 3 Department of Psychology, Liverpool John Moores University, Liverpool, UK

**Keywords:** Food insecurity, Academic performance, Higher education, University students, GPA, College food insecurity

## Abstract

**Purpose of Review:**

Food insecurity is a concern in Higher Education globally, negatively impacting academic performance, and limiting the potential and aspirations of vulnerable groups of students. Previous reviews have focused primarily on the prevalence of food insecurity in high-income countries. The current review aimed to provide a cross-national synthesis of quantitative evidence of the association between food insecurity and academic performance among students aged 18 years and older. A comprehensive search of six electronic databases was conducted for published peer-reviewed articles up to May 31, 2025. The Joanna Briggs Institute appraisal tools were used to provide a quality assessment of the studies, and evidence synthesis was achieved by a vote-counting approach.

**Recent Findings:**

Forty-seven studies were included; the majority were cross-sectional (*N* = 46) and conducted in the United States of America (USA) (*N =* 37). The overall weighted mean prevalence of food insecurity among higher education students across the studies was 44.03%. Evidence of a negative association between food insecurity and academic performance was reported in 43 studies (91.5%), comprising 42 cross-sectional and one longitudinal study (*N* = 176,947) (USA, Canada, Nigeria, Malaysia, Australia, Iceland & Jordan). The remaining four cross-sectional studies found no significant association between food insecurity and academic performance (USA, Mexico, Saudi Arabia).

**Summary:**

The relatively high prevalence rate reported, the potential setbacks to academic success, and the inconsistencies observed within and across countries suggest that more prospective and comparative studies are needed to elucidate the driving mechanisms of this relationship. This will inform the development of effective, socio-culturally sensitive interventions.

**Supplementary Information:**

The online version contains supplementary material available at 10.1007/s13668-026-00744-6.

## Introduction

Higher education is a critical driver of social mobility and economic development [1]. Food insecurity, defined as an economic and social condition of limited or uncertain access to adequate nutritious food [2], is of concern due to higher- than -national average prevalence rates reported among students (e.g., 33% vs. 13.5%, USA) [3, 4]. Similar stark disparities have also been observed in other countries such as Malaysia, Canada, and Nigeria [5–7]. Thus, food insecurity is not only disproportionately prevalent in higher education institutions, but cuts across both low- and high-income countries. With the introduction of equal opportunities and widening participation policies in most countries, the demographics of higher education students have drifted from the traditionally “affluential” or “elite” group to include under-represented groups such as the socioeconomically disadvantaged, ethnic minorities, mature students, and first-generation backgrounds [8, 9]. While these policies have diversified enrolment, and possibly advanced social justice, vulnerability of minority groups to other forms of structural inequality have also become apparent. Students from disadvantaged backgrounds often lack the economic and social resources of their more privileged counterparts, making them more likely to encounter food insecurity in an economically demanding academic environment [10].

The high prevalence of food insecurity among higher education student populations is particularly concerning due to its widely reported negative impact on academic outcomes. These include poor class attendance [11, 12], concentration difficulties [13, 14], diminished motivation [15], dropout ideation [10, 16], and low-grade point average (GPA) [17–19]. GPA is a widely used metric for assessing academic performance in higher education in most countries, particularly the USA. When compared to food-secure counterparts, Haro-Contreras et al. [17] and Raskind et al. [18] found approximately a 0.14-point decrease in GPA attributable to students’ food insecurity status. Similarly, Hege et al. [20] reported that food-insecure students were nearly six times more likely to achieve below the minimum GPA compared to food-secure students. Given the potential impact of food insecurity on GPA, and the use of GPA as a proxy for work-related cognitive and non-cognitive competencies by employers [21], food insecurity may hinder students’ future social mobility and economic prospects, including post-graduation employability and earnings in the labour market. In studies exploring the impact of undergraduate GPA on employability prospect, GPA of applicants was found to significantly predict success in securing a job [22], average salary [23], and long-term career success [24].

However, despite several studies reporting a negative association, others have found no significant difference in academic performance by food insecurity status [25–27]. A systematic examination of available data could provide a more nuanced understanding of the experiences of students and how the discrepancy has arisen. Also, previous systematic reviews on food insecurity in higher education have primarily focused on prevalence and in specific regions (e.g., high-income countries), with inconclusive secondary evidence on the association with academic performance [28, 29].

Bruening et al. [28] reviewed both peer-reviewed and grey literature from a range of countries and reported a 35%–42% prevalence of food insecurity among students, and its association with adverse academic outcomes such as lower GPA (13 out of 58 studies). McKay et al. [29], on the other hand, conducted a meta-synthesis of 156 studies from seven high-income countries and reported a pooled food insecurity prevalence of 42.2% and a negative association with academic outcomes in 36 of the 38 studies that reported on academic outcomes. However, the search strategies and scope of both reviews present fundamental limitations regarding the evidence on the association between food insecurity and academic performance. First, because the primary focus of the reviews was on food insecurity prevalence and not on its direct association with academic performance, the general search strategies did not include academic performance or outcomes. This may have led to the exclusion of studies that provided evidence on the food insecurity–academic performance relationship. Second, the restriction to, or predominance of, evidence from high-income countries (e.g., USA, Canada, Australia) failed to capture the vital broader global socio-cultural and economic determinants of food insecurity, particularly in low- and middle-income countries.

The present review addressed these gaps by systematically synthesising evidence on the relationship between food insecurity and academic performance, drawing on studies from a wide range of global socio-cultural contexts. Expansion of the evidence base not only helps in the generalisability of findings, but assists in moving towards a consensus regarding the nature of the food insecurity-academic performance relationship to inform future research (e.g., exploring the mechanisms driving the relationship) and to guide the design of tailored interventions that respond to the diverse demographic characteristics of students.

## Methods

The systematic review followed the guidelines of the 2020 Preferred Reporting Items for Systematic Reviews and Meta-Analysis (PRISMA) statement [30].

### Eligibility Criteria

The inclusion criteria were set a priori using the Population, Exposure, Comparator, Outcome, and Study (PECOS) framework [31]. *Population*: undergraduate or postgraduate university or college students of any country aged 18 years and above. *Exposure*: food insecurity, measured by validated international or country-specific instruments. *Comparator*: food-secure students. *Outcomes*: academic performance measured by grade point average (GPA), letter grades, percentage scores, or any standard measure of academic performance. *Study type*: published (English language) peer-reviewed journal articles of quantitative or mixed methods studies that reported effect size estimates either in the form of odds ratio (OR), correlation coefficient, regression coefficient, mean difference, or Chi-square test.

### Search Strategy

PubMed, PsychInfo, EbscoHost (Academic Search Complete, Education Search Complete, and ERIC), and Google Scholar databases were comprehensively searched for articles from inception until May 31, 2025 using the search term: (“food insecur*” OR “food secur*” OR “food poverty” OR “food insufficienc*” OR “food access” OR “food deprivation” OR hunger OR hungry OR starvation OR “food supply”) AND (“university student*” OR “college student*” OR “higher education” OR undergraduate* OR “undergraduate student*”) AND (“academic achievement*” OR “academic performance” OR “academic outcome*” OR “academic success” OR “academic attainment” OR “education* outcome*” OR “education* achievement*” OR “education* performance*” OR “education* attainment” OR “education* success” OR “education* failure” OR “academic failure” OR “grade point average” OR gpa OR “cumulative weighted average” OR cwa OR “weighted grade point average” OR wgpa). A manual search was conducted for additional studies by reviewing the reference lists of the included studies. All database searches and manual reference searches were conducted and logged by the first author.

### Selection Process

**Fig. 1:**
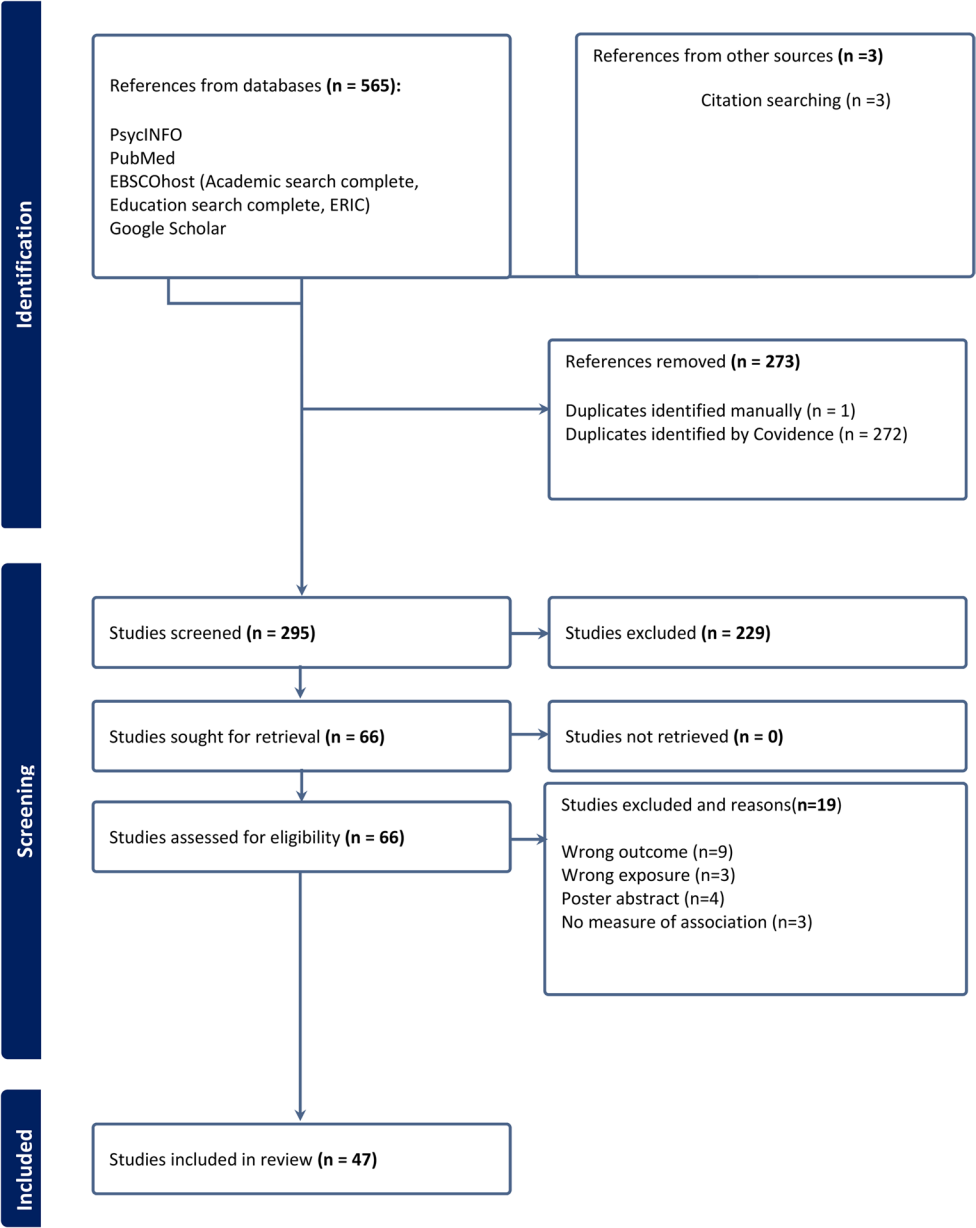
PRISMA (2020) flow diagram for record identification and selection for a systematic review of the association between food insecurity and academic performance of higher education students

The identified records from the database searches were imported to Mendeley Reference Manager and subsequently to Covidence (a web-based platform for systematic reviews) for deduplication, screening and selection. Eligible studies were independently screened by three reviewers. Titles and abstracts were independently screened and studies that passed the title and abstract screening were forwarded for full-text review to ensure all eligibility criteria were met. Full-text reviews were also performed independently by the reviewers and decisions made for inclusion. Where a study was excluded, reasons were given (Fig. 1). In the case of conflicting decisions on a study, the three reviewers resolved it by consensus.

### Data Extraction Process

After identifying all eligible studies, data extraction was completed by the first author and corroborated by the second author. Data extracted included author(s), publication year, year/month of data collection, study design, sample size and sample characteristics (i.e., population, country, institution, gender, average age, etc.), sampling and recruitment method, food insecurity measurement tool, main findings, correlates of food insecurity, and the statistical measure of association used, for a qualitative synthesis.

### Quality Assessment of Studies

The 8-item critical appraisal checklist for analytical cross-sectional studies from the Joanna Briggs Institute (JBI) was applied to assess the quality of the included cross-sectional studies [32]. The checklist assessed eight factors of a study, including, the definition of the inclusion criteria of the sample; the description of the participants and setting; the measurement of exposures, conditions, and outcomes; the identification and strategies to address confounding factors; and the appropriateness of the method of statistical analysis. For the longitudinal study in this review, the 11-item version for cohort studies was used for quality assessment. The additional items included in this checklist are the assessment of the changes over time; the sufficiency of the length of the follow-up period; the loss to follow-up; and the appropriateness of the strategies to address the loss to follow-up. The possible score for each item was “yes”, “no”, “unclear”, or “not applicable”. To rate the quality of the evidence as good, fair, or poor for each study, we adapted the following criteria from Shi et al., [33]: good (all “yes” or “not applicable” ratings), fair (1 to 2 “no” or “unclear” ratings), and poor (3 or more “no” or “unclear” ratings).

### Synthesis of Evidence and Analytical Strategy

The association between food insecurity and academic performance was categorised into three groups: (1) Negative (-): food insecurity was significantly associated with reduced academic performance; (2) Positive (+): food insecurity was significantly associated with improved academic performance; (3) Null association: there was no significant association between food insecurity and academic performance. Due to the methodological heterogeneity between studies, it was not appropriate to meta-synthesise the data altogether or on the subcategories of evidence of association. Therefore, evidence synthesis was done by a vote-counting method, an alternative approach suitable for heterogeneous evidence and used by Cope and Chestnutt [34]. The vote-counting approach weighed the evidence that showed a negative relationship between the independent and dependent variables against those that showed a null and positive association.

## Results

The database searches yielded 565 references, with an additional three studies identified through reference list searching of included articles. After deduplication, 295 references were screened by title and abstract. Following this screening, 66 articles were forwarded for full-text review against the eligibility criteria, resulting in the exclusion of 19 articles. A total of 47 articles were included for data extraction (Fig. 1).

### Study Populations

The studies were conducted in nine countries with the majority from the USA (*N* = 37). Two studies each were conducted in Canada [6, 35] and Malaysia [5, 36], and one each in Australia [37], Iceland [38], Mexico [25], Saudi Arabia [26], Nigeria [7], and Jordan [39] (Table 1). Except for two studies that were in 2-year community colleges [40, 41], all studies were in 4-year universities or colleges. Most studies recruited participants within the traditional college/university age range (18–24 years) [42]. The mean age of the participants across the twenty-two studies that reported it ranged from 20.1 ± 2.5 SD [43] to 23.6 ± 6.7 SD years [44]. Except for Ali et al. [39], female participants were the majority in all the studies, with a participation rate ranging from 50.6% [45] to 87% [46]. Most studies reported participants’ racial or ethnic backgrounds, with White students comprising the majority in 31 of the 47 studies. Twenty-four studies involved only undergraduate students, one study involved only graduate students [39], and twenty-two involved both undergraduate and graduate students. Although none of the studies reported on the socioeconomic status (SES) as a construct, the majority reported on proxies including respondent/household income (*N* = 14), employment status (*N* = 29), financial aid eligibility/receipt (*N* = 14) and parents’ educational level (*N* = 4).

**Table 1:** Characteristics and findings of included studies (*N=47*) in a systematic review of the association between food insecurity and academic performance among higher education students

First Author’s Initials, Year, Country	Study design/Sampling method	Population, Setting Sample size	FI measurement tool/Reference time	FI prevalence (%)	Measure of academic performance	Evidence/Type of association
ANSS, 2021 & 2022, Malaysia	C-SS/MPS	2nd, 3rd year UG *N* = 663	USDA 6-item 12 months	62.8	GPA	Yes (negative)
AT, 2025, USA	C-SS/PS	UG, 4-year college, New York City *N* = 246	USDA 10-item 3 months	20.6	GPA	Yes (negative)
AA, 2024, Jordan	C-SS/PS	PG, public university *N* = 126	USDA 10-item (adapted to 8 items) 12 months	8.0	GPA	Yes (negative)
BM, 2023, USA	C-SS/RS	UG, public university *N* = 314	USDA 6-item 12 months	47.9	GPA	Yes (negative)
BNA, 2024, Mexico	C-SS/PS	UG/PG of health programmes, public university *N* = 466	15-item ECLSA scale 3 months	47.0	AG	No (Null)
BN, 2023, Australia	C-SS/CS	UG and transitional course students, public university *N* = 664	USDA 6-item Throughout students’ studies	25.4	WAM	Yes (negative)
CK, 2019, USA	C-SS/RS	UG, public midsize university, Western US *N* = 3245	USDA 6-item 12 months	23	GPA	Yes (negative)
CM, 2020, USA	C-SS/PS	Nursing UG, public university *N* = 55	USDA 6-item 12 months	60	GPA	Yes(negative)
CC, 2022, USA	C-SS/RS	UG, public university *N* = 338	USDA 10 & 18-item 30 days	47	GPA	Yes (negative)
DR, 2021, USA	C-SS/NR	UG/PG, public university *N* = 1767	USDA 6-item N/R	46.8	GPA/Letter grade	Yes(negative)
EZA, 2019, USA	C-SS/CS	1 st year UG, 8 public universities *N* = 855	USDA 10-item 12 months	19	GPA	Yes(negative)
FNF, 2023, Malaysia	C-SS/CS	UG, 20 public universities *N* = 300	USDA 10-item During Covid-19 period	69	GPA	Yes (negative)
FK, 2025, Canada	C-SS/RS	UG/PG, public university *N* = 1694	USDA 6-item 30 days	46.6	GPA	Yes(negative)
FL, 2019, Canada	C-SS/CS	UG, public university *N* = 1030	USDA 10-item (adapted) 12 months	38.1	Letter grades	Yes(negative)
GME, 2023, USA	C-SS/PS	Social work UG/PG, public university *N* = 125	USDA 6-item 12 months	46.0	GPA	Yes(negative)
HR, 2019, USA	C-SS/CS	UG/PG, 10 public universities, *N* = 13,642	USDA 10-item 12 months	30.5	GPA and APS	Yes (negative)
HR, 2018, USA	C-SS/CS	UG/PG, rural public university *N* = 692	USDA 10-item 12 months	36.6	GPA and APS	Yes (negative)
HRH, 2024, USA	C-SS mixed method/RS	UG/PG, public university *N* = 2,**116**	USDA 10-item 12 months	16	GPA	Yes (negative)
HC, 2025, USA	C-SS/CS	UG self-identified Latina, Latino, or Latinx students, public university *N* = 1861	USDA 6-item 12 months	64	GPA	Yes (negative)
HC, 2023, USA	C-SS/RS	UG, public university *N* = 1087	USDA 6-item 12 months	36.0	GPA	Yes (negative)
HM, 2021, USA	C-SS/RS	UG/PG, public university. *N* = 1330	USDA 10-item 30 days	15.49	GPA	Yes (negative)
HA, 2020, USA	C-SS/CS	UG/PG, public university *N* = 1632	USDA 6-item 12 months	43	GPA	Yes (negative)
IE, 2024, Nigeria	C-SS/RS	UG, 18 private, federal and public universities *N* = 392	FCS N/R	60.0	GPA	Yes(negative)
KR, 2024, Saudi Arabia	C-SS/CS	UG/PG, public university *N* = 388	FIES 30 days	40.0	GPA/APS	No (Null)
MC, 2022, USA	C-SS/RS	UG/PG, 75 public universities *N* = 48,103	USDA 6-item 12 months	58.3	GPA and letter grades	Yes (negative)
MM, 2015, USA	C-SS/CS	UG, 2 community colleges *N* = 301	USDA 10-item 12 months	56	GPA	Yes (negative)
MS, 2020, USA	C-SS/RS	UG/PG, public universities *N* = 8705	USDA 6-item 12 months	40	GPA and letter grade	Yes (negative)
MH, 2024, USA	C-SS/RSS	UG/PG, public university *N* = 2654	USDA 10-item 30 days	31.7	Attrition rate and credit hour loss	Yes (negative)
MLM, 2016, USA	C-SS/RS	UG, 4 public universities *N* = 1882	USDA 10-item 9 months	35	GPA	Yes (negative)
MEM, 2025, USA	C-SS/RS	UG/PG, public university *N* = 2020	USDA 6-item 12 months	61	Concentration difficulty, thinking of delaying degree and delayed degree	Yes (negative)
ONM, 2017, USA	C-SS/PS	UG/PG food pantry users, rural public university *N* = 65	USDA 6-item 30 days	72.3	GPA	Yes(negative)
PLM, 2014, USA	C-SS/CS	UG/PG, rural public university *N* = 354	USDA 6-item N/R	59.0	GPA	Yes (negative)
PSD, 2018, USA	C-SS/CS	UG, public university *N* = 237	USDA 18-item 12 months	15.0	GPA	No (Null)
PE,2018, USA	C-SS/RS	UG, public university *N* = 508	USDA 6-item 12 months	36.6	GPA	Yes (negative)
RIG, 2018, USA	LS/RS	UG/PG, 7 public and private colleges/universities *N* = 2377	USDA 6-item 12 months	29	GPA	Yes (negative)
RBM, 2024, Iceland	C-SS/CS	UG/PG, 3 public universities *N* = 924	FIES 12 months	17.0	Perceived impact of food insecurity on academic performance	Yes(negative)
RRA, 2021, USA	C-SS/CS	UG, private urban university *N* = 257	USDA 6-item 12 months	36.6	GPA	No (Null)
SS, 2024, USA	C-SS/RS	UG campus food pantry users, public university *N* = 1170	USDA 6-item Spring academic term	62	GPA	Yes (negative)
TJJ 2022, USA	C-SS/SRS	UG, community college *N* = 238	USDA 10-item 12 months	52	GPA	Yes (negative)
TK, 2024, USA	C-SS/CS	UG/PG, public university *N* = 418	USDA 6-item 12 months	32	Missed class/study session/exam, dropped a class, and not performed to fullest academic potential	Yes (negative)
UM, 2023, USA	C-SS/PS	UG, public university *N* = 360	USDA 10-item 30 days	19	GPA	Yes(negative)
VI, 2023, USA	C-SS/PS	UG, public university *N* = 607	USDA 6-item 12 months	34	GPA	Yes(negative)
VWI, 2018, USA	C-SS/CS	UG, public university *N* = 591	USDA 6-item 1 month	59	GPA and retention	Yes(negative)
WRR, 2019, USA	C-SS/CS	UG, public university *N* = 2055	USDA 10-item 30 days	48	GPA	Yes (negative)
WR, 2018, USA	C-SS/CS	UG/PG, public university *N* = 4842	USDA 10-item 12 months	36	GPA	Yes(negative)
ZB, 2025, USA	C-SS/RS	UG/PG, 75 public and private colleges and universities, *N* = 63,680	USDA 6-item 30 days	40.8	GPA	Yes (negative)
ZVA, 2021, USA	C-SS/RSS	UG, public university *N* = 919	1 question, adapted from USDA-18 item 12 months	31.1	GPA	Yes(negative)

*AG* (Academic Grading), *AOR* (Adjusted Odds Ratio), *APS* (Academic Progression Score), *CS* (Convenience sampling), *C-SS* (Cross-sectional studies), *ECLSA* (Latin American and Caribbean Food Security Scale, acronym in Spanish), *FCS* (Food Consumption Score), *FI* (Food insecurity), *FIES* (Food Insecurity Experience Scale), *GPA* (Grade Point Average), *LS* (Longitudinal studies), *MPS* (Multi-stage purposive sampling), *N* (sample size), *N/R* (Not reported), *OR* (Odds Ratio), *PG* (Postgraduates), *PS* (Purposive sampling), *r-coefficient* (Pearson’s coefficient of correlation), *RS* (Random sampling), *SRS* (Stratified random sampling), *UG* (Undergraduates), *USDA* (United States Department of Agriculture), Weighted Average Mark (WAM)

### Study Designs and Sampling Strategies

Cross-sectional survey study designs predominated, with only one longitudinal study [18]. Two cross-sectional studies assessed food insecurity prevalence across two timelines of a student’s enrolment [47, 48]. The longitudinal study and twelve of the cross-sectional studies (e.g. [25, 49, 50]). were secondary analyses of data from broader parent projects. Three studies used a mixed-method cross-sectional approach [51–53].

The sample size of studies ranged from 55 [46] to 63,680 [54], with the majority (*N* = 30) drawing their sample from a single university. The largest study involved 75 institutions [54].

Convenience (*N* = 19), simple random (*N* = 15) and purposive (*N* = 10) sampling were the most used methods, and stratified random sampling the least used (*N* = 3). Emails to students was the predominant recruitment strategy, either together with or independent of other strategies such as class announcements, interception in public spaces, posters or flyers, social media, campus information booths and events, and food pantry records. Data collection lasted an average of four weeks. The response rate ranged from 6.08% [20, 52] to 87% [14].

### Quality Assessment

Most studies (*N* = 35) fully met the assessment criteria and were rated as “good” quality. This included the longitudinal study and thirty-four cross-sectional studies. The remaining studies were assessed as being of “fair” quality, primarily due to insufficient clarity on whether confounding factors were identified and appropriately controlled (e.g. [14, 16, 20, 36, 55]). The results of the quality assessment for each study are presented in *Online Resource 1*.

### Measurement of Food Insecurity

The majority of studies (*N* = 43) used the USDA Food Security Survey Module (FSSM) or its adapted version to determine food insecurity status. Of the three versions (i.e., the 6-item, 10-item, and 18-item), the 6-item version was the most used (*N* = 24). The other instruments included the Food and Agriculture Organisation’s (FAO) Food Insecurity Experience Scale, Food Consumption Score, and the Latin America and Caribbean Food Security Scale. The reference periods of measurement ranged from 12 months (*N* = 28) to 30 days or one month (*N* = 9). There were unconventional reference periods, such as 9 months, throughout the student’s stay in university, during the COVID-19 pandemic, and during the most recent academic term. Two studies did not report the reference period [49, 56].

### Measurement of Academic Performance

Students’ academic performance was primarily measured using standard grading metrics, including GPA, weighted average mark (WAM), academic grading (AG), letter grades, or a combination of these. The most commonly used metric, either independently or alongside other measures, particularly by studies conducted in the USA was GPA (*N* = 40). The Australian WAM was used by Brownfield et al. [37], while Betancourt-Núñez et al. [25] used the Mexican AG system. Other measures included the academic progression score (APS) [26, 57, 58], student attrition and credit hour loss [11], difficulty concentrating in class or delayed degree completion [13], and missed classes/exams or withdrawal from studies [51].

### Prevalence of Food Insecurity

Food insecurity prevalence among the study populations varied by institution but consistently exceeded national estimates, ranging from 8% to 72.3%, yielding an overall weighted mean prevalence of 44.03% across all studies. Among USA studies (*N* = 37) only, the weighted mean prevalence of 46.42% was about four times the national rate of 13.5% [4]. Weighted mean prevalence among Canada and Malaysia-based studies was 43.4% and 64.7%, respectively. Single-study estimates were 8% (Jordan), 17% (Iceland), 25.4% (Australia), 40% (Saudi Arabia), 47% (Mexico), and 60% (Nigeria).

Food insecurity prevalence also varied by the type of USDA assessment tool used. Studies that used the 6-item scale yielded a higher weighted mean prevalence (46.4%) compared to those that used the 10-item scale (32.4%). Studies with multiple timepoint analysis showed that food insecurity rates increased over the study duration. For instance, Van Woerden et al. [48] observed an increase from 37% in the Fall 2015 semester to 38% in the Spring 2016. Cuite et al. [47] also reported a near doubling of food insecurity prevalence from 22.4% in 2016 to 41.0% in 2019.

Food insecurity prevalence also tended to be higher among minority groups, such as students from non-White racial backgrounds (e.g. [40, 59–61]), Pell Grant recipients (e.g. [10, 60]., users of campus food assistance programmes such as food pantries (e.g. [18, 19, 56]), first-generation college students (e.g. [45–47]), and students who worked more hours (e.g. [20, 49, 59]).

### Evidence of Association between Food Insecurity and Academic Performance

Forty-three (91.5%), comprising 42 cross-sectional and one longitudinal study (*N* = 176,*947*), reported an inverse association between food insecurity and academic performance/outcomes. Specifically, 38 studies reported association with GPA, and the remaining reported associations with WAM (1), letter grades (1) and perceived impact (3). Most were conducted in the USA (*N* = 35), and the rest in Malaysia (2), Australia (1), Iceland (1), Nigeria (1), Jordan (1), and Canada (2). Four studies-USA [27, 43], Saudi Arabia [26], and Mexico [25]-found no significant association. No study reported a positive association (Table 1). Among studies that reported significant findings, 35 were rated as “good” quality. *Online Resource 2* presents details of the key findings from each study.

## Discussion

The review examined the association between food insecurity and academic performance. The majority of studies cross-nationally reported higher than average national prevalence rates and reduced academic performance in students experiencing food insecurity (e.g. [10, 17, 18, 62]). The findings suggested that food-insecure students were more likely to fall within the lowest GPA categories, and often trailed their food-secure counterparts in the odds of being in an upper GPA category even if they had the academic potential to do well (e.g. [5, 18–20, 40]).

The adverse association identified in this review extends the inconclusive secondary evidence from previous reviews. Bruening et al. [28] and McKay et al. [29], in their reviews of food insecurity among higher education students in predominantly high-income countries, both noted negative associations between food insecurity and academic outcomes, including GPA. While their reviews provided important preliminary insights into the food insecurity–academic performance relationship, the geographical reach and the general search strategy used limited the conclusiveness of the evidence. The present review addressed this limitation through a systematic and focused synthesis of evidence on the food insecurity–academic performance relationship from more diverse socio-cultural and economic contexts, spanning North America, Asia, Africa, and Europe. A key strength of the evidence is the inclusion of a longitudinal study that examined the prospective effect of food insecurity on academic performance. The study showed a consistent pattern of negative association between food insecurity and GPA across multiple time points. Although not causal evidence, the replication of findings over time strengthens the evidence for an adverse relationship and also points to the potential long-term impact of food insecurity on academic performance.

Beyond the association with GPA, the present review supported further findings of Bruening et al. [28] and McKay et al. [29], that food insecurity is linked with other poor academic outcomes, including poor concentration in class, absenteeism, lost credit hours, neglected studies, delayed degree completion and withdrawal [11, 12, 14]. These food insecurity-induced academic disruptions not only help to explain the negative food insecurity-GPA association observed in our review, but also underscore the broader, far-reaching ramifications of food insecurity on students’ academic engagements. Students experiencing food insecurity may be particularly vulnerable to these disruptions due to the physical manifestations of food insecurity such as fatigue [63]; strain of financial struggles [10]; psychological distress [18, 64]; and the trade-off between securing food and focusing on academics [63]. These observations also possibly suggest that food insecurity may still affect other forms of academic outcomes even when grades are not affected, reinforcing the argument by Payne-Sturges et al. [27] for the adoption of more comprehensive assessment metrics of academic performance, beyond single metrics such as GPA, when examining the academic impacts of food insecurity.

Although the present review found evidence for an adverse association, the use of general population food insecurity measurement tools across studies raises concerns about the accuracy of the prevalence estimates reported, particularly as none of the instruments have been validated for use in higher education settings. Nikolaus et al. [65] evaluated the psychometric properties of the predominantly used 6-item and 10-item versions of the USDA FSSM among university students and reported significant variation in food insecurity prevalence estimates, with the shorter version yielding higher estimates. This was also noticed in the present review, where studies that used the 6-item scale yielded a higher weighted prevalence. On the contrary, however, McKay et al. [29] reported that the prevalence estimate of the 10-item item scale was rather higher. While the two scales are reported to have a high agreement [65, 66], it remains unclear why they estimate food insecurity rates differently. These inconsistencies underscore the need for a validated food insecurity measurement tool for higher education students, to accurately estimate its prevalence within this population.

These methodological variations may also help to explain why some studies did not detect an association between food insecurity and academic performance in the present review. Four studies [25–27, 43] found no association, despite some participants acknowledging perceived academic impacts [25, 27]. This is consistent with the findings of a one-year prospective study among middle school adolescents that food insecurity was unrelated to students’ grades but predicted poorer academic skills [67]. In the case of the studies in the current review, the authors suggested that the effect or relationship may have been obfuscated by factors such as small sample size [43], the limitations with GPA [27], self-reporting biases [25], and participants’ potential resilience to food insecurity [26]. However, a critical appraisal suggests that additional study-specific factors may also be implicated. For example, apart from Ryan et al. [43], the other three studies used food insecurity measurement tools that are not consistently used in higher education food insecurity research. The use of the household-level USDA 18-item module and the 15-item ECLSA scale by these studies may have limited the accuracy of food insecurity prevalence estimates, particularly given the length and context. Nikolaus et al. [65] noted that the most widely used instruments in higher education food insecurity research are the USDA 6- and 10-item scales, with the latter providing the highest accuracy for identifying students with food insecurity. That three of the studies that found no effect were also the only studies in the review that used these instruments suggests a plausible explanation for their results.

In addition, restricted sampling frames and limitations in analytic strategies may have contributed to the null findings. Betancourt-Núñez et al. [25] recruited only students enrolled in health programmes, potentially limiting variability in socioeconomic background and academic potential, while Kahtan et al. [26] did not control for confounding variables in their analysis. Both sample homogeneity and confounders are known to reduce the likelihood of detecting effects in population studies [68]. Finally, unlike the majority of the included studies, Ryan et al. [43] was the only study conducted in a private urban university, which may have different socio-demographics from a public university.

Overall, evidence from the present review indicates an adverse association between food insecurity and academic performance among higher education students. However, methodological variations across studies may have contributed to inconsistencies in individual findings.

### Limitations

There are some limitations which should be considered when interpreting the findings. First, the review included only peer-reviewed, English-language journal articles, excluding grey literature and studies published in other languages. This may have resulted in the omission of relevant evidence from countries or regions underrepresented in the review. Second, due to methodological heterogeneity across studies, a vote-counting approach was employed for evidence synthesis rather than a meta-analysis. While vote counting allows for a directional comparison of findings, it does not provide information on the strength of associations and does not account for differences in study quality or sample size. Finally, qualitative studies were excluded to preserve the potential for meta-analytical synthesis. This decision limited the ability to capture students’ lived experiences and contextual factors surrounding food insecurity in the review.

### Implications for Future Research, Practice and Policy

There is a paucity of evidence outside North America, including longitudinal studies examining the prospective effects of food insecurity. For example, only a single European study met the inclusion criteria for this review, which is in contrast to the substantial contribution from North America. This may reflect differences in research priorities and/or differences in social welfare systems. The social policy configuration of a country has been shown to predict food insecurity risk. Berkowitz et al. [69] reported that countries with more robust welfare state regimes such as corporatist (e.g., Germany) and social democratic (e.g., Norway) models have significantly lower food insecurity risk than liberal welfare regimes like the USA. Therefore, more research attention is needed in countries with limited evidence to enable comparisons of the unique driving and protective factors of food insecurity across regions. Longitudinal studies will help to elucidate both the long-term impacts and the mechanistic pathways linking food insecurity to academic performance.

There is also a need for a validated food insecurity measurement tool for higher education populations. The widely used USDA (FSSM), validated for the general adult population, may not adequately capture fully the experiences of food insecurity among students. The instrument was developed for household settings and does not take into account the unique living circumstances of students, many of whom live away from home.

Higher education institutions are in a strategic position to help address and mitigate the impact of food insecurity on students. While food pantries and voucher schemes are the most commonly cited interventions, Hickey et al. [70] identified that multi-faceted approaches are more likely to succeed. Therefore, food insecurity support programmes could extend beyond food provision to include components such as financial management, budgeting, and nutrition education.

Establishing institutional systems that allow for students to voluntarily disclose their need for food insecurity support is essential for timely intervention, in addition to national policies that address the broader structural determinants of food insecurity.

## Conclusion

This review provides sufficient evidence that food insecurity is highly prevalent among higher education students and is associated with poor academic performance. Addressing student food insecurity is critical to ensuring that all students have equitable opportunities to fulfil their academic potential and achieve their desired place on the social mobility ladder. Further prospective research on the driving mechanisms of this relationship is needed to inform both national and institutional-level interventions to tackle and mitigate the impact of food insecurity on students’ wellbeing and educational success.

## Key References

- McKay FH, Olajide BR, Melleuish LJ, Pitt P, Lau EH, Dunn M. Food insecurity among post-secondary students in high income countries: systematic review and meta-analysis. *Curr Nutr Rep.* 2025;14(1):58. 10.1007/s13668-025-00651-2.
  - ○ This paper presents evidence from 156 studies on food insecurity among post-secondary students in high-income countries, highlighting its high prevalence and adverse academic and health outcomes. It represents a comprehensive and up-to-date meta-analysis on food insecurity in higher education.
- Berkowitz SA, Drake C, Byhoff E. Food insecurity and social policy: a comparative analysis of welfare state regimes in 19 countries. Int J Soc Determin Health Health Serv. 2024;54(2):76–86.10.1177/27551938231219200.
  - ○ This cross-national comparative study provides evidence linking welfare-state regimes to food insecurity risk, highlighting how social policy configurations shape population vulnerability and resilience. Its findings offer critical policy context for understanding the structural drivers of food insecurity across countries.
- Brownfield N, Quinn S, Bates G, Thielking M. What is eating Gilbert’s grades? Examining the impact of food insecurity and psychological distress on weighted average marks within a sample of Australian university students. *J Further High Educ.* 2023;47(5):659–73.10.1080/0309877X.2023.2176203.
  - ○ This study demonstrates both direct and indirect associations between food insecurity and the academic performance of university students. It is one of the few studies to identify a mechanistic pathway-psychological distress-through which food insecurity adversely affects students’ academic outcomes.

## Supplementary Information

Below is the link to the electronic supplementary material.

Supplementary Material 1 (PDF 242 KB)

Supplementary Material 2 (PDF 279 KB

## Data Availability

No datasets were generated or analysed during the current study.
