# Supplementary material for: The Association between Food Insecurity and Academic Performance among Higher Education Students: A Systematic Review
Source: Curr Nutr Rep. 2026 Feb 28;15(1):17. doi: 10.1007/s13668-026-00744-6 (PMC12950061; doi:10.1007/s13668-026-00744-6)
Supplement: Supplementary file 1 — Supplementary Material 1 (PDF 242 KB) [file 13668_2026_744_MOESM1_ESM.pdf]

Title: The association between food insecurity and academic performance among higher education students: A systematic review.

Journal: Current Nutrition Reports

Table S1 Quality assessment of included studies (*N=47*) in a systematic review on the association between food insecurity and academic performance.

**First Author's Initials, Year, Country**	**Question 1**	**Question 2**	**Question 3**	**Question 4**	**Question 5**	**Question 6**	**Question 7**	**Question 8**	**Question 9**	**Question 10**	**Question 1 1**	**Overall Rating [c]**
**Cross-sectional studies [a]**												
ANSS, 2021&2022 Malaysia	Yes	Yes	Yes	N/A	Yes	Yes	Yes	Yes	N/A	N/A	N/A	Good
AT, 2025, USA	Yes	Yes	Yes	N/A	Yes	Yes	Yes	Yes	N/A	N/A	N/A	Good
AA, 2024, Jordan	Yes	Yes	Yes	N/A	Unclear	Unclear	Yes	Yes	N/A	N/A	N/A	Fair
BM, 2023, USA	Yes	Yes	Yes	N/A	Unclear	Unclear	Yes	Yes	N/A	N/A	N/A	Fair
BNA, 2024, Mexico	Yes	Yes	Yes	N/A	Yes	Yes	Yes	Yes	N/A	N/A	N/A	Good
BN, 2023, Australia	Yes	Yes	Yes	N/A	Yes	Yes	Yes	Yes	N/A	N/A	N/A	Good

CK, 2019, USA	Yes	Yes	Yes	N/A	Yes	Yes	Yes	Yes	N/A	N/A	N/A	Good
CC, 2022, USA	Yes	Yes	Yes	N/A	Yes	Yes	Yes	Yes	N/A	N/A	N/A	Good
CM, 2020, USA	Yes	Yes	Yes	N/A	Yes	Yes	Yes	Yes	N/A	N/A	N/A	Good
DR, 2021, USA	Yes	Yes	Yes	N/A	No	No	Yes	Yes	N/A	N/A	N/A	Fair
EZA, 2019, USA	Yes	Yes	Yes	N/A	Yes	Yes	Yes	Yes	N/A	N/A	N/A	Good
FNF,2023, Malaysia	Yes	Yes	Yes	N/A	No	No	Yes	Yes	N/A	N/A	N/A	Fair
FK, 2025, Canada	Yes	Yes	Yes	N/A	Yes	Yes	Yes	Yes	N/A	N/A	N/A	Good
FL, 2019, Canada	Yes	Yes	Yes	N/A	Yes	Yes	Yes	Yes	N/A	N/A	N/A	Good
GME, 2023, USA	Yes	Yes	Yes	N/A	Yes	Yes	Yes	Yes	N/A	N/A	N/A	Good
HR, 2019, USA	Yes	Yes	Yes	N/A	Yes	Yes	Yes	Yes	N/A	N/A	N/A	Good
HR, 2018, USA	Yes	Yes	Yes	N/A	Yes	Yes	Yes	Yes	N/A	N/A	N/A	Good
HRH, 2024, USA	Yes	Yes	Yes	N/A	Yes	Yes	Yes	Yes	N/A	N/A	N/A	Good
HC, 2025, USA	Yes	Yes	Yes	N/A	Yes	Yes	Yes	Yes	N/A	N/A	N/A	Good
HC, 2023, USA	Yes	Yes	Yes	N/A	No	No	Yes	Yes	N/A	N/A	N/A	Fair
HM, 2021, USA	Yes	Yes	Yes	N/A	Yes	Yes	Yes	Yes	N/A	N/A	N/A	Good

HA, 2020, USA	Yes	Yes	Yes	N/A	Unclear	Unclear	Yes	Yes	N/A	N/A	N/A	Fair
IE, 2024, Nigeria	Yes	Yes	Yes	N/A	Yes	Yes	Yes	Yes	N/A	N/A	N/A	Good
KR, 2024, Saudi Arabia	Yes	Yes	Yes	N/A	No	No	Yes	Yes	N/A	N/A	N/A	Fair
MC, 2022, USA	Yes	Yes	Yes	N/A	Yes	Yes	Yes	Yes	N/A	N/A	N/A	Good
MM, 2015, USA,	Yes	Yes	Yes	N/A	Yes	Yes	Yes	Yes	N/A	N/A	N/A	Good
MS, 2020, USA	Yes	Yes	Yes	N/A	Yes	Yes	Yes	Yes	N/A	N/A	N/A	Good
MH, 2024, USA	Yes	Yes	Yes	N/A	Yes	Yes	Yes	Yes	N/A	N/A	N/A	Good
MLM, 2016, USA	Yes	Yes	Yes	N/A	Unclear	Unclear	Yes	Yes	N/A	N/A	N/A	Fair
MEM, 2025, USA	Yes	Yes	Yes	N/A	Yes	Yes	Yes	Yes	N/A	N/A	N/A	Good
ONM, 2017, USA	Yes	Yes	Yes	N/A	No	No	Yes	Yes	N/A	N/A	N/A	Fair
PLM, 2014, USA	Yes	Yes	Yes	N/A	Yes	Yes	Yes	Yes	N/A	N/A	N/A	Good
PSD, 2018, USA	Yes	Yes	Yes	N/A	Yes	Yes	Yes	Yes	N/A	N/A	N/A	Good
PE, 2018, USA	Yes	Yes	Yes	N/A	Yes	Yes	Yes	Yes	N/A	N/A	N/A	Good
RBM, 2024, Iceland	Yes	Yes	Yes	N/A	Yes	Yes	Yes	Yes	N/A	N/A	N/A	Good
RRA, 2021, USA	Yes	Yes	Yes	N/A	Yes	Yes	Yes	Yes	N/A	N/A	N/A	Good

SS, 2024, USA	Yes	Yes	Yes	N/A	Yes	Yes	Yes	Yes	N/A	N/A	N/A	Good
TJJ, 2022, USA	Yes	Yes	Yes	N/A	No	No	Yes	Yes	N/A	N/A	N/A	Fair
TK, 2024, USA	Yes	Yes	Yes	N/A	Yes	Yes	Yes	Yes	N/A	N/A	N/A	Good
UM, 2023, USA	Yes	Yes	Yes	N/A	Yes	Yes	Yes	Yes	N/A	N/A	N/A	Good
VI, 2023, USA	Yes	Yes	Yes	N/A	No	No	Yes	Yes	N/A	N/A	N/A	Fair
VWI, 2018, USA	Yes	Yes	Yes	N/A	Yes	Yes	Yes	Yes	N/A	N/A	N/A	Good
WR, 2019, USA	Yes	Yes	Yes	N/A	Yes	Yes	Yes	Yes	N/A	N/A	N/A	Good
WR, 2018, USA	Yes	Yes	Yes	N/A	Yes	Yes	Yes	Yes	N/A	N/A	N/A	Good
ZB, 2025, USA	Yes	Yes	Yes	N/A	Yes	Yes	Yes	Yes	N/A	N/A	N/A	Good
ZVA, 2021, USA	Yes	Yes	Yes	N/A	No	No	Yes	Yes	N/A	N/A	N/A	Fair
**Longitudinal studies [b]**												
**First Author's Initials, Year, Country**	**Question 1**	**Question 2**	**Question 3**	**Question 4**	**Question 5**	**Question 6**	**Question 7**	**Question 8**	**Question 9**	**Question 10**	**Question 11**	**Overall Rating**
RIG, 2018, USA	Yes	Yes	Yes	Yes	Yes	N/A	Yes	Yes	Yes	Yes	Yes	Good

[a]The following 8 questions were used for the cross-sectional studies:

Question1. Were the criteria for inclusion in the sample clearly defined? Question 2: Were the study subjects and the setting described in detail? Question 3: Was the exposure measured in a valid and reliable way? Question 4: Were objective, standard criteria used for measurement of the condition? (Not applicable in this review) Question 5: Were confounding factors identified? Question 6: Were strategies to deal with confounding factors stated? Question 7: Were the outcomes measured in a valid and reliable way? Question 8: Was appropriate statistical analysis used?

[b]The following 11 questions were used for the longitudinal study: Question 1: Were the two groups similar & recruited from the same population? Question 2: Were the exposures measured similarly to assign people to both exposed and unexposed groups? Question 3: Was the exposure measured in a valid and reliable way? Question 4: Were confounding factors identified? Question 5: Were strategies to deal with confounding factors stated? Question 6: Were the groups/participants free of the outcome at the start of the study or at the moment of exposure? (This question was not applicable in this review). Question 7: Were the outcomes measured in a valid and reliable way? Question 8: Was the follow up time reported and sufficient enough for outcomes to occur? Question 9: Was follow up complete, and if not, were the reasons to loss to follow up described and explored? Question 10: Were strategies to address incomplete follow up utilized? Question 11: Was appropriate statistical analysis used?

[c]Overall rating: To rate the quality of the evidence as good, fair, or poor for each study, we used the following criteria: good (all 'yes' or 'not applicable' ratings), fair (1 to 2 'no' or 'unclear ratings'), and poor (3 or more 'no' or 'unclear' ratings) as adapted from (Shi et al., 2021)

[d]Unclear: if authors were not explicit on the item

[e]N/A: Not applicable
