# Supplementary material for: The Association between Food Insecurity and Academic Performance among Higher Education Students: A Systematic Review
Source: Curr Nutr Rep. 2026 Feb 28;15(1):17. doi: 10.1007/s13668-026-00744-6 (PMC12950061; doi:10.1007/s13668-026-00744-6)
Supplement: Supplementary file 2 — Supplementary Material 2 (PDF 279 KB [file 13668_2026_744_MOESM2_ESM.pdf]

Title: The association between food insecurity and academic performance among higher education students: A systematic review.

Journal: Current Nutrition Reports

Table S2 Key findings of included studies (*N=47*) in a systematic review on the association between food insecurity and academic performance among university and college students

First Author's Initials, Year, Country	Key findings	Correlates/Mediators/Moderators of food insecurity
ANSS, 2021 & 2022, Malaysia	1. Food insecurity is significantly associated with academic performance and psychosocial status.<br>2. Food insecure students less likely to get a pointer more than or equal to 3.7 for their academic performance compared to food-secure students (AOR = 0.363, 95%, CI:1.22-34.014).	***Correlates***: Financial aid, parent occupational status, and monthly household income.<br><br>***Mediator:*** Psychosocial (depression & anxiety) status.
AT, 2025, USA	1.Students with food insecurity reported a lower GPA (z = −2.87, p=.004, Cohen's d=.442), and were more likely to report that food issues interfered with their academics (z=8.68, p<.001, Cohen's d=1.30).<br><br>2. Compared to students with food security, students with food insecurity had lower household income (z = −2.98, p=.003, Cohen's d=.495), were more likely to use food pantries (z=3.00, p=.003, Cohen's d=.482), and to receive food assistance from religious organisations (z=2.46, p=.014, Cohen's d=.390) and	***Correlates:***<br><br>Race**,** household income, immigrant generational status,

	were marginally more likely to use SNAP (z=1.96, p=.0502, Cohen's d=.356)	
AA, 2024, Jordan	1.The level of food security is inversely associated with academic performance (GPA).<br><br>2. The average GPA of food secure students was 12% higher than that of food insecure students (91% vs79%).	***Correlates:***<br><br>Family size, marital status, employment status, and income
BM, 2023, USA	1. Students who experienced food insecurity had lower GPAs, were twice as likely to fail and withdraw from a course and were four times more likely to take an incomplete grade in a course.<br><br>2. The mean GPA of students with food insecurity was .30 points lower than students who did not experience food insecurity [MD=0.30(t = -6.184, p<0.001)]	***Correlates:*** Failing a course, withdrawal from a course, and taking an incomplete course
BNA, 2024, Mexico	1. Food insecurity not significantly (r=-0.080, p > 0.05) correlated with Academic Grading.<br><br>2.Moderate & severe food insecurity significantly associated with perceived overall progress in college (OR: 2.96; 95%CI: 1.49, 5.88) and attendance to classes (OR:3.14; 95%CI: 1.19, 8.28) as poor or regular, and positively related to perceiving difficulties in completing their studies (OR:2.75; 95%CI: 1.43, 5.29).<br><br>3. Depression, anxiety, stress, and sleep quality are not significantly associated with Academic Grading.	***Correlates:***<br><br>Age, gender, employment status, anxiety, stress and depression.
BN, 2023, Australia	1.Food insecurity was associated with poorer academic performance and increased psychological distress. Both the direct and indirect effects of FI on academic performance were	***Correlates:*** Renting privately, having a disability, receiving government benefit, and age (being older).

	significant and negative (Direct effect = −.09 and Indirect effect= -0.03, p < .05)	***Mediator:*** Psychological distress
CK, 2019, USA	1. FI was negatively associated with GPA (r= –.18, p < .01) and partially mediated the relationship between students' sociodemographic characteristics and GPA	***Correlates:***<br>Race (being Black and Hispanic), Pell grant recipient, first generation status, more part time work hours
CM, 2020, USA	1. GPAs differed statistically significantly between food-secure and food-insecure students [MD=0.15(t=2.16, p=0.3)]	***Correlates:***<br>First generation status and social support (significant others, family, and friends)
CC, 2022, USA	1.There was a significant relationship between food security status and GPA even after controlling for the demographic covariates.<br>That is, food insecurity at either time point (2016 & 2019) was related to a lower GPA in 2019 [F (7,325) = 5.74, P < 0.001] and that the mean GPA of food insecure students was lower than their comparators at either time point, at least by 0.24 (SE = 0.07, p<0.001) points.	***Correlates:***<br>First generation status and age.
DR, 2021, USA	1.Students with lower GPAs were much more likely to be food insecure than those with higher GPAs (p < .001). For example, 75% of students with a GPA equivalent grade of D/F were food insecure compared to 42.4% of those with a GPA equivalent grade A. 3.<br>2.Food insecure students were statistically significantly more likely to also experience challenges with academics (p < .001, phi = 0.18), careers (p < .001, phi = 0.15), procrastination (p < .001,	***Correlates:***<br>Mode of study, level of study, race, year of study and number of part-time hours.

	phi = 0.13), and faculty (p = .001, phi = 0.08) when compared to food secure counterparts.	
EZA, 2019, USA	1.Food security status was significantly associated with self-reported GPA (p = 0.001).<br><br>2.After controlling for socio demographic characteristics, food insecure students were almost twice the risk of having a GPA < 3.00 compared to food-secure students [OR = 1.91(95% CI: 1.19 - 3.07)].	***Correlates:***<br><br>Race (minority), off-campus residence, Pell grant recipient, parental education status of parents (high school or less), and meal plan (not having a meal plan), poor sleep quality, high stress, disordered eating.
FNF, 2023, Malaysia	1. FI was significantly associated with the cumulative GPA of students [χ 2 (1, n=300) = 7.739, p = 0.021].	***Correlates:***<br><br>Working status of parents(father), monthly income, and financial aid eligibility
FK, 2025, Canada	1.Food insecure students were less likely to report a GPA of 'A' (67.8 vs. 57.9%), and more likely to report a GPA of 'B', 'C' or 'D' (41.9 vs. 32.2%), χ2 (13) = 20.2, p < .001, V = .113.<br><br>2.Food insecure students were significantly less likely to indicate a sense of belongingness to their university (84.5 vs. 92.2%), χ2 (5) = 30.1, p < .001, V = .136, that their university placed priority on health and well-being (69.2 vs. 77.2%), χ2 (5) = 28.8, p < .001, V = .132<br><br>3. students deemed food insecure were rated significantly higher in psychological distress (M = 15.75; SD = 9.38 vs. M = 10.79; SD = 7.64), t (1605) = 11.69, p < .001, d = −.365	***Correlates:***<br><br>Sex, degree level, fee status, family income
FL, 2019, Canada	1.Moderately food insecure students were more than twice [AOR=2.57 (1.56–4.23)] as likely to report overall grades in the C,	***Correlates:***

	D, and F range than food-secure students, while severely food insecure students were over five times as likely to report poorer grades [AOR=5.84 (3.14–10.84)]	Living arrangements, having a meal plan, the primary source of income used to pay for educational costs, working status, and year of study
GME, 2023, USA	1.There were statistically significant group differences in GPA between food insecure (M=3.56) and food secure (M=3.81) students [t (123) =3.87, p<.001].<br><br>2.Most of this difference was attributed to students experiencing very low food security having the lowest average GPA (M=3.36), F (2,122) =16.20, p<.001.	***Correlates:***<br><br>Loan status, accommodation type, employment status, household composition, level of degree, filing as FAFSA
HR, 2019, USA	1.Lower GPA was associated with increased severity of food insecurity status [OR=0.73 (CI=0.60-0.87].<br><br>2.Food-secure students exhibited better academic performance as represented by APS scores and higher GPAs (mean differences were significant for both constructs)- MD=0.20 ±11, p<0.0001	***Correlates:***<br><br>Year of study, ethnicity, financial aid, health status, cooking frequently, and university.
HR, 2018, USA	1. The odds of high APS scores were inversely related to food insecurity [OR = 0.79; 95% CI 0.73- 0.86)].<br>2. Food insecurity status also showed significant differences in GPA as average GPA of food-insecure students was 3.33 ± 0.03 and average GPA of food secure students was 3.51 ± 0.02 (p < 0.0001)	***Correlates:***<br><br>Health status, housing status (off- campus residency), and year of study.
HRH, 2024, USA	1.Food insecurity was associated with having a GPA of less than 3.0-students with a GPA of less than 3.0 were around 2.6 times more likely to have a low food security status than students with a GPA of between 3.5 to 4.0 (p<0.001).<br><br>2. Mental health was also strongly associated with food access and security. Students who reported being depressed or having	***Correlates:***<br><br>Disability status, fee status, race, first generation status, living arrangement

	limited control over their life were around 2 or 6 times, respectively, more likely to have a low food security status when compared with students who reported no concerns	
HC, 2025, USA	1.There was a negative association between food insecurity and academic achievement; that is, students who report being food insecure also reported a decline in GPA (β=−0.134, p<0.001).<br><br>2. Food insecurity was associated with increased depression and anxiety symptomatology. For every point increase in the food insecurity scale, depression increased by .218 meanwhile anxiety by .175.<br><br>3.The use of food pantry campus resources was associated with an increase in academic GPA (β=0.058, p<0.01) and a decline in mental health symptoms (depression: β=-0.061, p<0.01; and anxiety: β=-0.053, p<0.05)<br><br>4. However, the moderation effect of immigration status and food pantry in the relationship between food insecurity and (i) mental health and (ii) academic achievement was not significant.	***Correlates:***<br><br>Immigration status, gender, economic status,
HC, 2023, USA	1. Food-insecure students had a significantly lower GPA (p < 0.001), were more likely to be non-white (p < 0.0001), and were more likely to have received financial aid compared to food-secure students (p < 0.0001).<br><br>2.Significant differences in GPA were found based on food security status (t (1039) = 34.78; p < 0.001) as food-insecure students had a significantly lower GPA compared to food-secure students (3.36 ± 0.42 vs. 3.50 ± 0.36).	***Correlates:***<br><br>Race, campus food pantry usage, employment status, volunteering, financial aid reception, first generation status, government assistance (SNAP)

HM, 2021, USA	1.Food insecurity in the prior month was associated with lower GPA, lower psychological well-being, and fewer hours of sleep.<br><br>2. Psychological well-being and sleep mediated the link between food security and GPA<br><br>3. The food secure student was 30 times more likely to have a GPA in the 3.5 - 4.0 range rather than a GPA below 2.5 than the food insecure student whose odds of being in the same higher GPA category is 6.846.	***Correlates:***<br><br>NR<br><br>***Mediators:***<br><br>Well-being and sleep
HA, 2020, USA	1.Food insecure students were nearly seven times more likely to have a lower cumulative GPA (less than 2.5) as compared to their food secure counterparts [OR=6.7 (CI=2.5-17.6)].<br><br>2. Food insecure students had poorer health status, difficulty concentrating on their studies, and suspended their studies more often.<br><br>3. More than a third of food insecure students did not purchase all the required course materials and 11% had to suspend their studies due to finances.	***Correlates:***<br><br>Financial aid, childhood food insecurity, income, employment status, working more hours, and housing insecurity.
IE, 2024, Nigeria	1.For both categories of students (poor and nonpoor), the direct effect of food insecurity on academic performance (CGPA) was significant (ß -0.424, p<0.0001 and ß= - 0.223, p<0.0001) respectively.<br><br>2. Mental stress mediated the relationship between food insecurity and academic performance (ß =-0.405, p<0.0001).	***Correlates:***<br><br>Age, health related habits, level of study, sponsors income, engagement in economic activities, parental monthly income.<br><br>***Mediators:***<br><br>Mental stress

KR, 2024, Saudi Arabia	1.No significant correlation was found between food insecurity and academic performance (r=-0.67, p=0.269).<br><br>2. Significant associations were observed between food insecurity and monthly household income ($p < 0.001$) and general health ($p < 0.005$), respectively.	***Correlates:***<br><br>Income level<br><br>General health
MC, 2022, USA	1.Very low food security was inversely associated with high GPA as it decreases the odds of obtaining a grade A or higher GPA category by 41% compared to high or marginal food security [Regression coefficient(ß)= −.523, OR = .59, p < .001).<br><br>2.Low food security reduces the odds of obtaining a grade A or a higher GPA category by approximately 25% [Regression coefficient (ß)=−.289, OR= .75, p < .001].<br><br>3. Reduced food security was associated with worse mental health measures (psychological distress, loneliness, psychological wellbeing).	***Correlates:***<br><br>Race, year in school, residential status, visa status, psychological distress, loneliness, psychological wellbeing.
MM, 2015, USA	1.Food insecure students were more likely than food secure students to report a lower GPA (2.0–2.49) than a higher GPA (3.5–4.0) (ß= −0.941, OR-0.39, p= .042)	***Correlates:***<br><br>Residential status (living alone), Race(minority), and parental type (single parenthood).
MS, 2020, USA	1. Food insecurity was directly and indirectly related to lower student GPA (even after controlling for all covariates) [Standardised path coefficient (ß)=–0.08, and indirect effect=-0.09, p<0.001)	***Correlates:***<br><br>race, sex, financial aid, employment, level of study, campus affiliation.<br><br>***Mediator:***<br><br>Poor mental health

MH, 2024, USA	1.Food-insecure students were over 1.6 times as likely to withdraw from or fail multiple courses compared to food-secure students after controlling for ethnicity, gender and sexual identity.<br><br>2. Food-insecure students on average lost more credit hours due to course failures and withdrawals than food-secure students (i.e., an average of 1.31 credit hours compared to an average of 0.97 credit hours)	***Correlates:***<br><br>Ethnicity, gender, sexuality
MLM, 2016, USA	1.A Chi square test showed that there is a significant association between GPA and food security status. Students with the lowest GPA (0–1.99) were less likely to be highly food secure ($x2= 84.466$; $P < .001$).	***Correlates:***<br><br>Race (minority), financial status (loan use), and living location(off-campus)
MEM, 2025, USA	1.Low food security was associated with poor concentration among students. The odds of experiencing concentration difficulties were more than four times as high in the "low" food security group compared to that in the "marginal/high" food security group (OR = 4.342, 95% CI [3.219, 5.858]), and more than eleven times as high in the "very low" food security group compared to that in the "high" food security group (OR = 11.149, 95% CI [8.139, 15.274])<br><br>2. Both "low" and "very low" food security increased the likelihood of students thinking about extending their academic programs. In the "very low" food security group, the odds of "often thought about delay" were more than six times higher than that in the "marginal/high" food security group (OR = 6.560, 95% CI [4.779, 9.006]), and, in the "low" food security group, the odds were around twice as high (OR = 3.041, 95% CI [2.210, 4.185]).	***Correlates:***<br><br>Income level, degree level, race, employment status, accommodation type

	3. Students with "very low" food security were significantly more likely to delay their degree completion compared to those with "marginal/high" food security (OR = 4.820, 95% CI [3.175, 7.319])	
ONM, 2017, USA	1.Food security scores and GPA were negatively correlated [r (62) = -.32, p = .01]. That is, as food security scores rose, indicating more food insecurity, GPA scores fell.<br><br>2. There were no statistically significant relationships between the second two sets of variables (i.e., food insecurity & health, and health & academic performance.	***Correlates:*** N/R
PLM, 2014, USA	1 Good academic performance (grade point average of greater than or equal to 3.1) was inversely associated with food insecurity as students with a grade point average of greater than or equal to 3.1 were 60% less likely to be food insecure [OR= 0.40; 95% (CI, 0.22–0.69)].	***Correlates:***<br><br>Income, health status (fair or poor), having employment, and participation in a food assistance programme (SNAP).
PSD, 2018, USA	1.There was no statistically significant difference in self-reported GPA by food security status(p=0.39).<br><br>2.Food insecure and at-risk students were in poorer health than food secure students as they were more likely to report fair, poor, or very poor and reported lower energy levels compared with food secure students.<br><br>3. Food insecure students reported more frequent depression symptoms and that they experienced disruptions in academic work because of depression symptoms.	***Covariates:***<br><br>Age, gender, family income<br><br>***Correlates:***<br><br>Race (African American), financial aid(beneficiaries), residential status (off-campus residents), health status and depression.

PE,2018, USA	1.Food insecurity is associated with lower GPAs, such that being food insecure is associated with a 0.17 points lower GPA than food secure students [Regression coefficient (ß) =−0.174, SE=0.064, p<0.01].<br><br>2. Food insecure students had 3.42 times greater odds of reporting neglecting their academic studies due to the money they owed.<br><br>3. Students who were employed part time or full time also had greater odds<br><br>of neglecting their studies (odds ratios 3.51 and 4.64, respectively).<br><br>4. Students with experiences of food insecurity had 3.49 times greater odds of<br><br>reporting that they had considered dropping out of college due to the money they owed than their food secure counterparts.	***Correlates:***<br><br>Race, first generation status, financial independence, parenting status (i.e., Childcare responsibilities), residential status.
RIG, 2018, USA	1.In adjusted linear regression models, food insecurity was associated with a 0·14-point decrease in GPA (SE=0·04, P=0·002), a 1·83-point increase in depression score (SE=0·25, P<0·0001), a 3·68-point increase in anxiety score (SE=0·69, P <0·0001), and a 2·16-point decrease in hope score (SE=0·36, P <0·0001).<br><br>2. In the final SEM, food insecurity was associated (standardised β, SE) with poorer psychosocial health (0·22, 0·03, P <0·0001) and poorer psychosocial health was associated with a lower GPA (−0·21, 0·03, P<0·0001)<br><br>3. The total effect of food security status on GPA was significant (standardised β= − 0·06, SE=0·02, P =0·002).	***Correlates:***<br><br>Race, employment status, SNAP benefit status, parental educational status, adverse childhood events.<br><br>***Mediator:***<br><br>Psychosocial health

	4.The indirect effect of food security status on GPA, as mediated by psychosocial health, was significant (standardised β = − 0·05, SE=0·01, P <0·0001) and accounted for 73 % of the total effect.<br><br>5. However, after accounting for psychosocial health, the direct effect of food security status on GPA was not significant (−0·02, 0·02, P=0·43).	
RBM, 2024, Iceland	1.After adjusting for gender and age, food-secure respondents were found to be less likely to have negative academic performance compared to food-insecure respondents [OR = 0.53, 95% CI (0.29, 0.91), p = 0.02]<br><br>2. Food-secure respondents had nearly twice the likelihood of experiencing positive or neutral academic performance compared to food-insecure respondents [OR = 1.94, 95% CI (1.09, 3.52), p = 0.02]	***Correlates:***<br><br>Change in food consumption and support network
RRA, 2021, USA	1. Food insecurity was not significantly associated with grade point average [AOR= 1.77 (0.67–4.70)].<br><br>2.Food insecurity was associated with lower perceived health status and greater consumption of sugar-sweetened beverages (SSBs).<br><br>2.Food insecurity was not associated with fruit and vegetable intake, alcohol consumption or sleep quality.	***Correlates*:**<br><br>Race, lower perceived health status, poor dietary intake (SSBs).
SS, 2024, USA	1.Food insecurity was negatively associated with GPA (ß =−0.18, 95% CI: −0.25−−0.10, p<0.001<br><br>2. Historically racialised status (HRS) and low socioeconomic position (SEP) students had significantly higher odds of experiencing food insecurity (OR = 2.72; 95% CI: 1.52–4.97) and	***Correlates:***<br><br>Gender, age, campus affiliation, financial status

	lower GPA (B = −0.14, p = 0.05) than traditional students, after adjustment	
TJJ 2022, USA	1.There was an association between being food secure and scoring a GPA of 3.5 or higher ($\chi 2 = 7.077$, $p = .008$).<br><br>2. Students' who self-reported to have a GPA between 3.5 to 4.0 were more likely to report being food secure when compared to other GPA groups.	**Correlates**:<br><br>Race
TK, 2024, USA	1.Food insecurity impacted academic and socioeconomic student behaviour- The biggest impact of food insecurity for food insecure respondents was missing a class and exam, dropping a class and not buying a required textbook.<br><br>2. 87% of the students who reported that food insecurity caused them to miss a class/exam, drop a class or not buy a required textbook were food insecure.	**Correlates:**<br><br>Race, sexual orientation, first generation status, residential category, mode of transportation
UM, 2023, USA	1.Food insecure students showed lower GPA [ß= 0.06 (0.01, 0.11)] and poor health compared to food secure students.<br><br>2.Students without FI had significantly greater GPA (median = 3.50 vs. 3.00) and lower depression (median = 8.00 vs. 15.50), anxiety (median = 41.00 vs 47.00), and pain interference score (median = 1.00 vs. 2.00) when compared to those with FI ($p \leq .05$<br><br>3. The link between FI and GPA was moderated by first-generation student status, with the negative impact of FI on GPA more clearly observed among non-first-generation students.	***Correlates***:<br><br>Age, sex, race, psychological health status (depression, anxiety) and physical health (bodily pain).<br><br>***Moderator:***<br><br>First generation status
VI, 2023, USA	1. Experiencing more food insecurity was related to lower GPAs ($r = -.17$, $p < .001$). However, this was moderated by race and	***Correlates:***<br><br>Income

	income as food insecurity was negatively related to grades for low-income White students only.<br><br>2. For all students, lower income was associated with more food insecurity (r =−.24, p < .001) and higher GPAs (r = .21, p < .001).	***Moderators:***<br><br>Race and income
VWI, 2018, USA	1. The GPA of food-insecure students was significantly lower than the GPA of food-insecure students in both the fall 2015 (3.00 vs. 3.25, p < 0.001) and spring 2016 (2.97 vs. 3.24, p = 0.003) semesters<br><br>2. Food-insecure students had a significantly lower GPA than food-secure students. In fall 2015, 59% of food-insecure students obtained at least a "B" grade (GPA = 3.00); this percentage would increase to 72% if these same students were food secure.<br><br>3. Food-insecure students were significantly more likely to work for pay than their food-secure counterparts in both the fall 2015 (31% vs. 21%; p = 0.012) and spring 2016 (51% vs. 37%; p = 0.009) semesters	***Correlates:***<br><br>Work for pay
WRR, 2019, USA	1. Food insecurity increased the odds of being among the lower 10% GPA and reduced the odds of being among the upper 10% GPA. Thus, when other variables are controlled for, food secure students have about three times the odds of being among the top group compared to food insecure students [OR= 0.34 (0.20–0.58)]<br><br>2. Being employed improves the odds of being among the top 10% for GPA (OR=1.78, CI: 1.03–3.07).	***Correlates:***<br><br>Ethnicity (black and Hispanics), year of study (second year students), financial aid (recipients), sex (female), meal plan beneficiaries, and commuters.

WR, 2018, USA	1. Students with a lower self-reported GPA (<3·85) were significantly more likely to be food insecure than students in the highest self-reported GPA range (3·85–4·00) [OR=2·85, 95% CI:2·23-3·6].<br><br>2. Students with previous food insecurity were 4·78 times more likely to be food insecure than the students who did not experience food insecurity before college.<br><br>3. Students who had part-time jobs were 1·28 times more likely to be food insecure compared with those who did not have part time jobs (95 % CI 1·10, 1·50).	***Correlates:***<br><br>Previous experience of FI, employment (part and full-time), familial financial support, and financial aid
ZB, 2025, USA	1.A correlation test showed a negative association between food insecurity and GPA, r = −0.14, p < .001.<br><br>2. Also, when controlling for other factors, food insecurity still emerged as a significant negative predictor of GPA, β =−0.10, p < .001<br><br>3.Food insecurity was positively associated with stress level (p<0.001)	***Correlates:***<br><br>Parents' educational level, student's financial status, smoking status, sexual harassment and discrimination
ZVA, 2021, USA	1. Food insecure students were found to be less successful in school with a mean GPA of 3.09 compared to 3.25 of food secure counterparts (MD=0.16, p <0.001).<br><br>2. Additionally, food insecurity was significantly associated with reduced ability to maintain focus during classes, p = 0.028, and less satisfaction with current academic performance, p =0 .001.<br><br>3. Reporting food insecurity was associated with having a depression diagnosis (22.4% vs. 14.3%), p = 0.005 and eating fewer than three servings of fruits and vegetables per day	***Correlates:***<br><br>lack of focus in class, dissatisfaction with current academic performance, depression, eating fewer than three servings of fruits and vegetables per day, poor sleep pattern, fair or poor self-rated health status,

	(14.4% vs. 7.3%), p <0.001, compared to those who were food secure.	
